# Reviewer acknowledgement 2014

**DOI:** 10.1186/s13098-015-0009-4

**Published:** 2015-02-20

**Authors:** Daniel Giannella-Neto

**Affiliations:** Universidade Nove de Julho, San Paola, Brazil

## Abstract

The Editors of *Diabetology & Metabolic Syndrome* would like to thank all of our reviewers, both external and Editorial Board Members, who have contributed to the journal in Volume 6 (2014) and whose valuable support is fundamental to the success of the journal.

**Rohana Abdul Ghani**

Malaysia

**Ludovico Abenavoli**

Italy

**Zeinab Abotalib**

Saudi Arabia

**Jamal Ahmad**

India

**Hasan Ahmed**

Canada

**Bo Ahrén**

Sweden

**Fulya Akin**

Turkey

**Mohammed Al**-**Gayyar**

Egypt

**Karriem Ali**

United States of America

**Bianca de Almeida**-**Pititto**

Brazil

**Andreia Alzamora**

Brazil

**Stephan Garcia Andrade**-**Silva**

Brazil

**Toby Andrew**

United Kingdom

**Jean**-**Luc Ardilouze**

Canada

**Benjamin Arnold**

United States of America

**Yoshimasa Aso**

Japan

**Anna Aulinas**

Spain

**Rosa Bacchetta**

Italy

**Luciana Bahia**

Brazil

**William Baker**

United States of America

**Jo Anne Balanay**

United States of America

**Samuele Baldasseroni**

Italy

**Francisco Bandeira**

Brazil

**Andrea Baragetti**

Italy

**Jerzy Bełtowski**

Poland

**Damasia Becu**-**Villalobos**

Argentina

**M. Fernanda Bellolio**

United States of America

**Jifeng Bian**

United States of America

**Antonino Bianco**

Italy

**Yochai Birnbaum**

United States of America

**Petter Bjornstad**

United States of America

**Francisco Bolaños**-**Jiménez**

France

**Chad Borges**

United States of America

**Eliete Bouskela**

Brazil

**Phillip Brantley**

United States of America

**Dionisios Bratis**

Greece

**Meryl Brod**

United States of America

**Jonathan Brooke**

United Kingdom

**Sonia Butalia**

Canada

**Eugene Butkowski**

Australia

**Salameh Bweir Al Dajah**

Jordan

**Jose Cabezas**-**Cerrato**

Spain

**Christian Cadeddu**

Italy

**Weibin Cai**

China

**Cleber Camacho**

Brazil

**Luis Henrique Canani**

Brazil

**Basar Candemir**

Turkey

**Per**-**Ola Carlsson**

Sweden

**Denise Carvalho**

Brazil

**Chiara Caselli**

Italy

**Ana Cenarro**

Spain

**Eugenio Cersosimo**

United States of America

**Wing Bun Chan**

Hong Kong

**Chu**-**Chih Chen**

Taiwan

**Harn**-**Shen Chen**

Taiwan

**Shih**-**Ann Chen**

Taiwan

**Jing Chen**

United States of America

**Yue**-**Hwa Chen**

Taiwan

**Wanchun Chiu**

Taiwan

**José Cipolla**-**Neto**

Brazil

**Paulo Collett**-**Solberg**

Brazil

**Robert Considine**

United States of America

**Ben Corden**

United Kingdom

**Maria Lucia Correa**-**Giannella**

Brazil

**Carlos Couri**

Brazil

**Mario Cruciani**

Italy

**Estela Cuevas**

Mexico

**Alberto Cuocolo**

Italy

**Jorge Daher Jr**.

Brazil

**Hiroyuki Daida**

Japan

**Cecilia Dall**'**Aglio**

Italy

**Kathy Dameron**

United States of America

**Allison Dart**

Canada

**Elisabeth de Jonge**

United States of America

**Adelzon de Paula**

Brazil

**Gareth Denyer**

Australia

**Ramakrishna Devaki**

India

**Laura Di Renzo**

Italy

**Carlos Dias Jr**.

Brazil

**Kenan Direk**

United Kingdom

**Hossam Draz**

Canada

**Ivone Duarte**

Brazil

**Daniel Duerr**

Germany

**Anastasia Efimenko**

Russian Federation

**Brendan Egan**

Ireland

**Samantha Ehrlich**

United States of America

**Samar El Khoudary**

United States of America

**Theodoros Eleftheriadis**

Greece

**Hassan El**-**Sayyad**

Egypt

**Diana Esposito**

United Kingdom

**Sandra Roberta Gouvea Ferreira**

Brazil

**María Luz Fika Hernando**

Spain

**Maelán Fontes Villalba**

Spain

**Maria Foss**-**Freitas**

Brazil

**Anna Ludovica Fracanzani**

Italy

**Edward Franek**

Poland

**Melissa Galea**

United Kingdom

**Daniel Gallaher**

United States of America

**Mingming Gao**

United States of America

**Xin Gao**

China

**Andrew Gardner**

United States of America

**Christopher Garrett**

United Kingdom

**Anca Gaston**

Canada

**Neoklis Georgopoulos**

Greece

**Laura Gianotti**

Italy

**Joga Gobburu**

United States of America

**Carlos Gois**

Portugal

**Ricardo Gomez**-**Huelgas**

Spain

**Miguel A. Gonzalez**-**Gay**

Spain

**Giuseppe Grosso**

Italy

**Nicola Guess**

United Kingdom

**Irina Gurieva**

Russian Federation

**Helene Hanaire**

France

**R. Luke Harris**

Canada

**Jeannine Heckmann**

South Africa

**John Heiker**

Germany

**Rainer Hellweg**

Germany

**Yasuhide Hibino**

Japan

**Yukihito Higashi**

Japan

**Karim Hmadcha**

Spain

**Tobias Hofmann**

Germany

**Elizabeth Holmes**-**Truscott**

Australia

**Sangmo Hong**

Korea, South

**Kuang Hongyu**

China

**Philip Hooper**

United States of America

**Mark Hopkins**

United Kingdom

**Christian Høst**

Denmark

**Jiung**-**Pang Huang**

Taiwan

**Jianying Huang**

United States of America

**Matthew Hudson**

United States of America

**Miguel Huerta**

Mexico

**Travis Hull**

United States of America

**Joseph Hung**

Australia

**Chung**-**Lieh Hung**

Taiwan

**Yousri Hussein**

Egypt

**Gianluca Lacobellis**

United States of America

**Avraham Ishay**

Israel

**Yasushi Ishigaki**

Japan

**Yoshio Iwashima**

Japan

**Reethi Iyengar**

United States of America

**Mark Jeschke**

Canada

**Jose Jimenez**

Costa Rica

**Tianru Jin**

Canada

**Steve Johnson**

Canada

**Jonathan Johnston**

United Kingdom

**Corinne Jolivalt**

United States of America

**Tadafumi Kajimoto**

Japan

**Sanjay Kalra**

India

**Nozomu Kamei**

Japan

**Nancy Kanagy**

United States of America

**Thomas Kapellen**

Germany

**Kaffer Kara**

Germany

**Ritu Karoli**

United States of America

**Ryuichi Kawamoto**

Japan

**Benjamin Kearns**

United Kingdom

**Hassan Khan**

United Kingdom

**Shokei Kim**-**Mitsuyama**

Japan

**Ken Kishida**

Japan

**Ana Kiss**

Brazil

**Noah Kolb**

United States of America

**Laszlo Koranyi**

Hungary

**Nana Kragh**

Denmark

**Tereza Kratzerova**

Czech Republic

**Puttarin Kulchaitanaroaj**

Thailand

**Masaaki Kurasaki**

Japan

**Alina Kurylowicz**

Poland

**Keisuke Kuwahara**

Japan

**Atsukazu Kuwahara**

Japan

**Hyo**-**Bum Kwak**

Korea, South

**Joan C Laguna**

Spain

**Jorma Lahtela**

Finland

**Lena Lämmle**

Germany

**Rodrigo Lamounier**

Brazil

**Maria Lankinen**

Finland

**Olga Laosa**

Spain

**Annunziata Lapolla**

Italy

**Terje Larsen**

Norway

**Douglas Lee**

Canada

**Hwa Lee**

United States of America

**Myung Gull Lee**

Korea, South

**Cristiane Leitao**

Brazil

**Andrea Leiva**

Chile

**Claudio Letizia**

Italy

**Siu**-**wai Leung**

Macao

**Wei**-**Dong Li**

China

**P. Andy Li**

United States of America

**Xin Li**

China

**Aris Liakos**

Greece

**Kae**-**Woei Liang**

Taiwan

**Jun Liang**

China

**Josivan Lima**

Brazil

**Ping**-**Ting Lin**

Taiwan

**Benigno Linares Segovia**

Mexico

**Christopher Lipina**

United Kingdom

**Randie Little**

United States of America

**Jun**-**Li Liu**

Canada

**Gary Lopaschuk**

Canada

**Jose Luchsinger**

United States of America

**Melinda Lull**

United States of America

**Michael Lustgarten**

United States of America

**Ruy Lyra**

Brazil

**Changsheng Ma**

China

**Jun Ma**

United States of America

**Ubiratan Machado**

Brazil

**Isabel Madeira**

Brazil

**Norikazu Maeda**

Japan

**Shiro Maeda**

Japan

**Dianna Magliano**

Australia

**Paolo Magni**

Italy

**Brij Mohan Makkar**

India

**Usman Malabu**

Australia

**Reija Männikkö**

Finland

**Edoardo Mannucci**

Italy

**Malgorzata Marchelek**-**Mysliwiec**

Poland

**Leticia Martin Cordero**

Spain

**Letizia Marullo**

Italy

**Marcelo Mascaro**

Brazil

**Tetsuya Matoba**

Japan

**Tandi Edith Matsha**

South Africa

**Morihiro Matsuda**

Japan

**Munehide Matsuhisa**

Japan

**Anselmo Mc Donald**

Panama

**Anita Mehta**

India

**Inês Meireles de Sousa**

Portugal

**Ricardo Meirelles**

Brazil

**Zaher Merhi**

United States of America

**Kotb Metwalley**

Egypt

**Yasuyoshi Miyata**

Japan

**Wataru Mizunoya**

Japan

**Mei Chung Moh**

Singapore

**Deepak Mohale**

India

**Regina Moises**

Brazil

**Babak Mokhlesi**

United States of America

**Maurizio Montella**

Italy

**Rodrigo Moreira**

Brazil

**Anselmo Moriscot**

Brazil

**Ryuichi Morishita**

Japan

**Shirin Jahan Mumu**

Bangladesh

**Kazufumi Nakamura**

Japan

**Shuhei Nakanishi**

Japan

**Yoshio Nakata**

Japan

**Carlos Negrato**

Brazil

**Armando Nevarez**-**Sida**

Mexico

**Denise Ney**

United States of America

**Leszek Niepolski**

Poland

**Mitsushiko Noda**

Japan

**Robert Noland**

United States of America

**Thomas Nyström**

Sweden

**Hisao Ogawa**

Japan

**Yoshihisa Okamoto**

Japan

**Julicristie Oliveira**

Brazil

**Erin Olson**

United States of America

**Altan Onat**

Turkey

**Yuri Ono**

Japan

**Nada Orsolic**

Croatia

**Eduardo Ortega**

Spain

**Richard Thomas Oster**

Canada

**Rytas Ostrauskas**

Lithuania

**Noriyuki Ouchi**

Japan

**Zeynel Abidin Ozturk**

Turkey

**Giuseppe Paolisso**

Italy

**Nazareno Paolocci**

United States of America

**Yong**-**Moon Park**

United States of America

**Mi Jung Park**

Korea, South

**Sun Ah Park**

Korea, South

**Sung**-**Ji Park**

Korea, South

**Patrizia Pasanisi**

Italy

**Francisco Pasquel**

United States of America

**Abel Pavìa**

Mexico

**Carlos Penha**-**Goncalves**

Portugal

**Maria Adelaide Albergaria Pereira Pereira**

Brazil

**Wilza Peres**

Brazil

**Manuel Pestana**

Portugal

**Mario Petretta**

Italy

**Errol Philip**

United States of America

**Olivia Phung**

United States of America

**Lars Pieper**

Germany

**Augusto Pimazoni**-**Netto**

Brazil

**Gustavo Duarte Pimentel**

Brazil

**Brigitte Pittet**-**Cuenod**

Switzerland

**Gilles Plourde**

Canada

**Mark Plummer**

Australia

**Alex Rafacho**

Brazil

**Ali Reza Rahbar**

Iran

**Asmah Rahmat**

Malaysia

**Pragna Rao**

India

**Mathias Rask**-**Andersen**

Sweden

**Nelson Rassi**

Brazil

**Gérard Reach**

France

**Andre Reis**

Brazil

**Sirimon Reutrakul**

Thailand

**Antonello Rigamonti**

Italy

**Vladimir Ritov**

United States of America

**Vittoria Rizzello**

Italy

**Eduardo Rodrigues**

Brazil

**Mariangela Rondanelli**

Italy

**James Rosado**

United States of America

**Rebecca Rosenwasser**

United States of America

**Francesco Rossi**

Italy

**Alberto F. Rubio**-**Guerra**

Mexico

**Justin Ryder**

United States of America

**Massimo Sacchetti**

Italy

**Pedro Saddi**-**Rosa**

Brazil

**Serenella Salinari**

Italy

**Josepa Salvado**

Spain

**Jonnathan Santillán**

Mexico

**Ferdinando Carlo Sasso**

Italy

**Warapone Satheannoppakao**

Tajikistan

**Nils Helge Schebb**

Germany

**Sallie Schneider**

United States of America

**Miranda Schram**

Netherlands

**Dara Schuster**

United States of America

**Peter Schwarz**

Germany

**George Schweitzer**

United States of America

**Lai**-**Chu See**

Taiwan

**Cristina Sena**

Portugal

**Irina Shabalina**

Sweden

**Baiju Shah**

Canada

**Yousef Shahin**

United Kingdom

**Gabriel Shaibi**

United States of America

**Rei Shibata**

Japan

**David Sigalet**

Canada

**Niina Susanna Siitonen**

Finland

**Satyesh Sinha**

United States of America

**Cynthia Smas**

United States of America

**Una Ørvim Sølvik**

Norway

**Wook Song**

Korea, South

**Sang**-**Wook Song**

Korea, South

**James Sowers**

United States of America

**Ragini Srivastava**

India

**Christiane Stabe**

Brazil

**Norbert Stefan**

Germany

**Bernd Stegmayr**

Sweden

**Anna M. Strømmen**

Denmark

**Charles Stuart**

United States of America

**Milton Suarez**-**Ortegón**

Colombia

**Naeti Suksomboon**

Thailand

**Grazyna Sypniewska**

Poland

**Mehrnoosh Tadayoni**

Iran

**Yohei Takai**

Japan

**Genzou Takemura**

Japan

**Zi**-**Hui Tang**

China

**Craig Taplin**

United States of America

**Gladys Teitelman**

United States of America

**Ozge Telci Caklili**

Turkey

**Alexander Tenenbaum**

Israel

**Solomon Tesfaye**

United Kingdom

**Thipaporn Tharavanij**

Thailand

**Anders Thorell**

Sweden

**Hao**-**ming Tian**

China

**Ramires Tibana**

Brazil

**Bogdan Timar**

Romania

**Heloisa Torres**

Brazil

**Curtis Triplitt**

United States of America

**Stefania Triunfo**

Spain

**Ivani Trombetta**

Brazil

**Apostolos Tsapas**

Greece

**Josep A. Tur**

Spain

**Faruk Turgut**

Turkey

**Lisa Tussing**-**Humphreys**

United States of America

**Antonino Tuttolomondo**

Italy

**Surendra Ugale**

India

**Jaime Uribarri**

United States of America

**Jose Lorenzo Valencia**-**Martin**

Spain

**Natalia Vallianou**

Greece

**Jana van Vliet**-**Ostaptchouk**

Netherlands

**Elizabeth Vaughan**

United States of America

**Orsolya Veber**

Hungary

**Sergio Vencio**

Brazil

**Marcio Vendramini**

Brazil

**Carolina Viana de Oliveira**

Brazil

**André Vianna**

Brazil

**Simone Vigod**

Canada

**Greisa Vila**

Austria

**Irene Vinagre**

Spain

**De Leo Vincenzo**

Italy

**Giovanni Vitale**

Italy

**Clemens von Schacky**

Germany

**Alisha Wade**

South Africa

**Christian Wadsack**

Austria

**Chih**-**Yuan Wang**

Taiwan

**Yu Wang**

Hong Kong

**Jim Waterhouse**

United Kingdom

**Jean Luc Wautier**

France

**Rebecca West**

United States of America

**Carol Westall**

Canada

**Jeong**-**taek Woo**

Korea, South

**Richard Woodman**

Australia

**Wanghong Xu**

China

**Yandiswa Yako**

South Africa

**Zhenyu Yang**

China

**Serbulent Yigit**

Turkey

**Dogan Yucel**

Turkey

**John Yudkin**

United Kingdom

**Roberto Luís Zagury**

Brazil

**Puhong Zhang**

China

**Yanglu Zhao**

United States of America

**Yan Zheng**

United States of America

**Linuo Zhou**

China

**Yimin Zhu**

China

**Bengt Zoller**

Sweden

**Petra Zürbig**

Germany

